# Effect of switching from twice-daily basal insulin to once-daily insulin glargine 300 U/mL (Gla-300) in Brazilian people with type 1 diabetes

**DOI:** 10.1186/s13098-024-01385-x

**Published:** 2024-07-09

**Authors:** Patricia Medici Dualib, Sergio Atala Dib, Gustavo Akerman Augusto, Ana Cristina Truzzi, Mauricio Aguiar de Paula, Rosângela Roginski Réa

**Affiliations:** 1 Escola Paulista de Medicina da Universidade Federal de São Paulo (EPM-Unifesp), São Paulo, Brazil; 2 CPQUALI Pesquisa Clínica, São Paulo, Brazil; 3 Sanofi, São Paulo, Brazil; 4 Serviço de Endocrinologia (SEMPR) do Hospital das Clínicas da Universidade Federal do Paraná (UFPR), Curitiba, Paraná Brazil; 5 Diabetes Center of the Endocrinology Division, Paulista School of Medicine - Federal University of São Paulo, Rua Estado de Israel 639, São Paulo, 04022-001 SP Brazil

**Keywords:** Diabetes mellitus, type 1, Insulin glargine, HbA1c, Glycemic profile, Hypoglycemia, Dawn phenomenon, Patient-reported outcomes

## Abstract

**Background:**

Low adherence to the number of insulin injections and glycemic variability are among the challenges of insulin therapy in type 1 diabetes (T1D). The TOP1 study investigated the effect of switching from twice-daily (BID) basal insulin to once daily (OD) insulin glargine 300 U/mL (Gla-300) on glycemic control and quality of life.

**Methods:**

In this 28-week, phase 4 trial, people with T1D aged ≥ 18 years, who were treated with BID basal insulin in combination with prandial rapid-acting insulin for at least 1 year, and had HbA1c between 7.5% and 10.0%, were switched to Gla-300 OD as basal insulin. The present study aimed to evaluate the impact of this change on HbA1c, glycemic profile, treatment satisfaction and safety. The change in HbA1c from baseline to Week 24 was the primary endpoint.

**Results:**

One hundred and twenty-three people with T1D (mean age 37 ± 11 years; 54.5% female) were studied. The disease duration was 20.0 ± 9.8 years, baseline HbA1c and fasting plasma glucose (FPG) were 8.6 ± 0.7% and 201 ± 80.3 mg/dL, respectively. After switching from BID to OD insulin regimen, no significant change in HbA1c was observed from baseline to Week 24 (*p* = 0.873). There were significant reductions in fasting self-monitoring blood glucose (SMBG) from baseline to Week 24 (175 ± 42 vs. 156 ± 38 mg/dL; *p* < 0.0001), and in glycemic profile (8-point SMBG) at several time points. There was a significant decrease in the proportion of patients with at least one hypoglycemic event (*p* = 0.025), in numbers of hypoglycemic events per patient-years of any type (*p* = 0.036), symptomatic (*p* = 0.007), and confirmed ≤ 70 mg/dL events (*p* = 0.049) from run-in to the last 4 weeks on treatment. There were significant improvements in treatment satisfaction (*p* < 0.0001), perceived hyperglycemia (*p* < 0.0001) scores and satisfaction with the number of injections between post-run-in and Week 24, and a significant decrease in fear of hypoglycemia.

**Conclusions:**

Switch from BID basal insulin to OD Gla-300 as part of basal *bolus* therapy in T1D resulted in similar glycemic control as measured by HbA1c, but provided significant improvements in SMBG, daily glucose profile, a lower incidence of hypoglycemia and increased patient satisfaction.

**Trial registration:**

NCT03406000.

**Supplementary Information:**

The online version contains supplementary material available at 10.1186/s13098-024-01385-x.

## Background

While type 1 diabetes (T1D) most frequently presents in childhood and adolescence, it is also diagnosed in adults, and the incidence in adults seems to be increasing in parallel to that in children [1, 2]. Insulin therapy in T1D is based on long- and short-acting insulins aiming good glycemic control and avoiding hypoglycemia and weight gain. Nevertheless, despite advances in this field, glycemic control in these patients remains suboptimal, with above-target HbA1c, high glycemic variability and sometimes with severe hypoglycemia episodes. There is a relationship between the type of the insulin regimen used and adherence to treatment [3]. Frequently, this becomes a limiting factor in the management of T1D [4]. In fact, fear of hypoglycemia and hypoglycemia events have a large impact on patient quality of life, and constitute a major obstacle for optimization of insulin therapy [5]. Reducing glycemic variability is an important factor that must be overcome during insulin treatment for T1D. In addition, reducing the number of daily injections may help overcome this potential barrier to treatment adherence, since the need for multiple daily insulin injections adds to the burden of T1D management. In this setting, the use of long-acting insulin analogues, when available, is more advisable than the use of classical human Neutral Protamine Hagedorn (NPH) insulin, as they provide less complex and more effective glycemic control with a reduced risk of hypoglycemia [6] and glycemic variability [7].

Two other important potential obstacles to optimal insulin treatment in T1D are the dawn phenomenon and the late-afternoon hyperglycemia. The dawn phenomenon is a term used to describe an increase in blood sugar or an increase in the amount of insulin needed to maintain normoglycemia during early morning hours [8]. Nearly half of the patients with T1D experience the dawn phenomenon [9]. Late-afternoon hyperglycemia may in part be the result of a tendency to better insulin action and lower hepatic insulin extraction at breakfast time than later in the day [10].

Insulin glargine 300 U/mL (Gla-300) is a second-generation, once-daily (OD) basal insulin analogue [11]. Gla-300 has a distinct pharmacokinetic and pharmacodynamic profile when compared with insulin glargine 100 U/ml (Gla-100) [12]. The higher concentration of Gla-300 originates a precipitate with a smaller surface area after subcutaneous injection compared with Gla-100, resulting in a steadier and extended glargine release, and leading to a smoother profile and longer duration of action (up to 36 h) [12, 13]. The present study aimed to evaluate the overall glycemic impact of switching from twice-daily (BID) basal insulin to OD Gla-300 in difficult-to-treat patients with T1D.

## Methods

### Study design

**Fig. 1:**
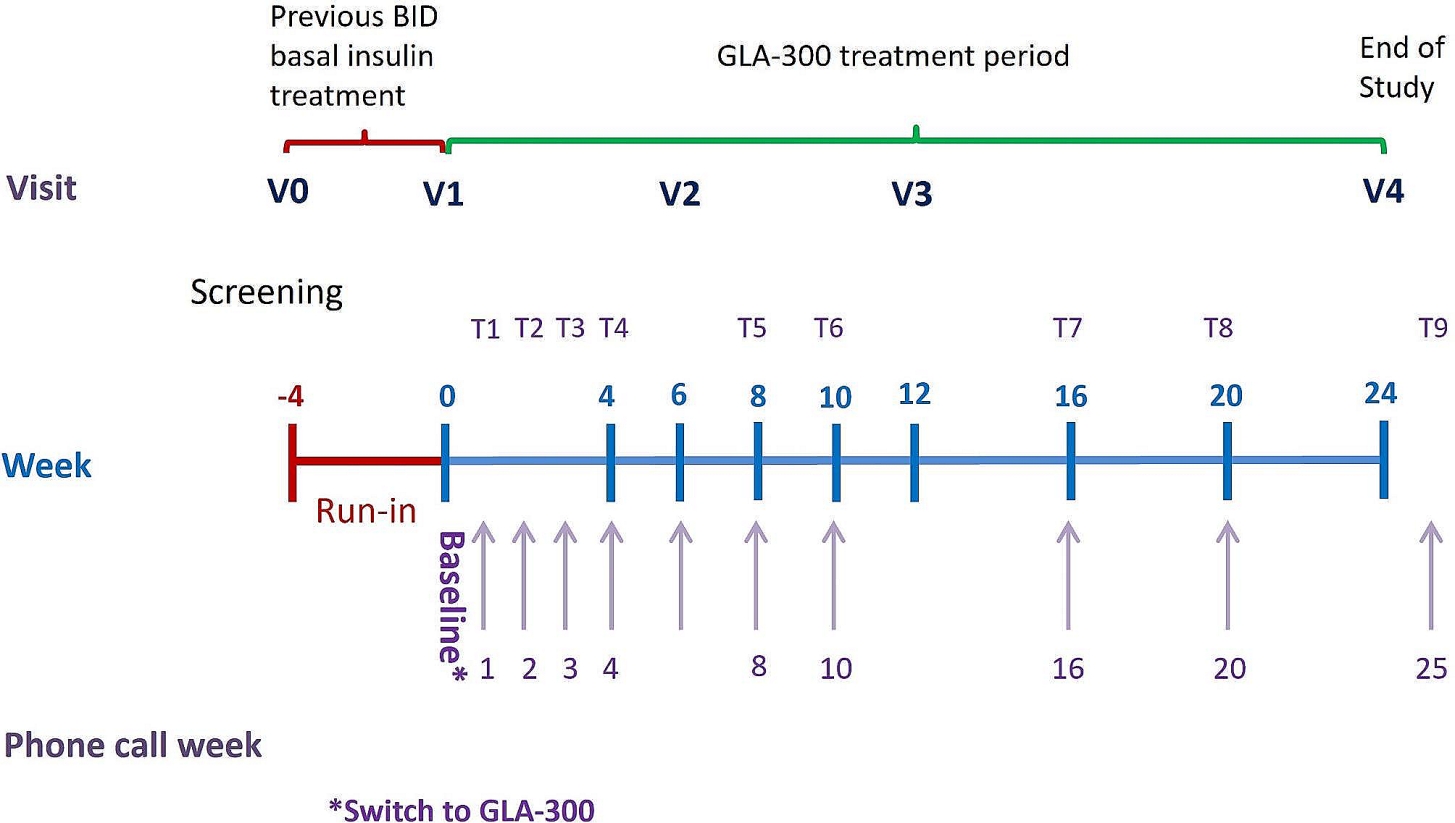
Study design

This was a 28-week, multicenter, prospective, interventional, single-arm, open-label phase 4 study conducted in Brazil. At screening, the selected eligible people with T1D entered a 4-week run-in period during which they continued their previous treatment without any additional intervention. Data on hypoglycemia events and self-monitoring of blood glucose (SMBG) were collected during this period to serve as control for the insulin treatment period. The study consisted of a 4-week run-in period, a 24-week treatment period when all patients received the same treatment (OD Gla-300 combined with their prandial insulin therapy) and underwent the same evaluations, and a 2–7 day safety follow-up period (Fig. 1). At the end of the run-in period, individuals who were still eligible according to the selection criteria were switched from their basal insulin treatment to OD Gla-300, continuing their prandial insulin therapy for the duration of the study, except if this treatment option had to be modified by the investigator for safety reasons.

The study was conducted after the approval by local ethics committee and in accordance with consensus ethics principles derived from international ethics guidelines, including the Declaration of Helsinki, and the International Conference on Harmonization guidelines for Good Clinical Practice. Informed consent was obtained from all patients prior to any study-related procedures.

### Patient eligibility

Eligible individuals were male or female, aged ≥ 18 years, had TD1 according to the American Diabetes Association criteria [14], were under treatment with any basal insulin BID in combination with any prandial rapid-acting insulin analogue for at least 1 year, had HbA1c between 7.5% and 10.0% at study entry, and signed an informed consent form. Exclusion criteria were type 2 diabetes mellitus; known hypoglycemia unawareness; repeated episodes of severe hypoglycemia or diabetic ketoacidosis within the last 12 months; end-stage renal failure or hemodialysis; any clinically significant abnormality identified on physical examination, laboratory tests, or vital signs at the time of screening or baseline; any major systemic disease resulting in short life expectancy that, in the opinion of the investigator, would restrict or limit the patient’s successful participation for the duration of the study; treatment with GLP1 agonists; participation in another clinical trial; any contraindications to the background therapies or warning/precaution of use (when appropriate) as displayed in the respective national product labeling; use of systemic glucocorticoids (excluding topical application or inhaled forms) for one week or more within 90 days prior to the time of screening; pregnancy or lactation; women of childbearing potential with no effective contraceptive method; known hypersensitivity/intolerance to insulin glargine or any of its excipients; and withdrawal of consent during the screening or run-in phase (including failure to return to the site).

### Study treatment and procedures

Gla-300 was to be administered before breakfast, between 6:00 and 10:00 AM. The injection time was defined at the start of the study, maintained as reference time for the whole duration of the study, and should be documented daily. By study protocol, the recommended starting dose of Gla-300 was as follows: (1) when switching from BID Gla-100 to Gla-300, the recommended starting OD dose was the same as the previous daily Gla-100 dose; (2) when switching from other BID basal insulin products to OD Gla-300, the recommended initial Gla-300 dose was 80% of the total daily dose of basal insulin agent that was discontinued. The dose of Gla-300 was adjusted to a target range according to fasting SMBG between 70 and 130 mg/dL (3.9–7.2 mmol/L) as per ADA 2015 Clinical Practice recommendations [14]. The algorithm for titration of Gla-300 is shown in Supplement Table 1. In case of a single fasting SMBG measurement below 3.9 mmol/L (below 70 mg/dL), it was recommended to decrease Gla-300 dose by 1 U.

**Table 1:** Selected patient characteristics at screening

Characteristic	Number (%) or Mean value (SD)
Gender (female)	67 (54.5%)
Age (years)	37 (11.5)
Age at diagnosis of diabetes^1^ (years)	17.0 (9.5)
Duration of diabetes^2^ (years)	20.0 (9.8)
Body weight (Kg)*	73.3 (13.2)
BMI (kg/m^2^)*	26.3 (4.1)
Systolic blood pressure^3^ (mmHg)	123.7 (12.8)
Diastolic blood pressure^3^ (mmHg)	77.2 (9.3)
Heart rate (beats per minute)	79 (9.6)
HbA1c (%)	8.6 (0.7)
FPG (mg/dL)**	201 (80.3)
BID basal insulin at screening	
Gla-100	77 (63%)
Detemir	28 (23%)
Isophane	16 (13%)
Degludec	2 (2%)
Basal insulin at baseline	
Gla-100	82 (67%)
Detemir	24 (19%)
Isophane	15 (12%)
Degludec	2 (2%)
History of any microvascular complications	46 (37.4%)
Retinopathy	34 (27.6%)
Neuropathy	13 (10.6%)
Nephropathy	18 (14.6%)
Diabetic foot	1 (0.8%)
History of macrovascular complications	
Hypertension	26 (21.1%)
Requiring pharmacological treatment	24 (19.5%)
Peripheral vascular disease	1 (0.8%)
Atrial fibrillation known	1 (0.8%)
TIA	1 (0.8%)
Angina pectoris without heart attack	2 (1.6%)
Hyperlipidemia/hypercholesterolemia	41 (33.3%)
Requiring pharmacological treatment	37 (30.1%)
Family history of stroke or coronary disease	42 (34.1%)

BMI, body mass index; FPG, fasting plasma glucose; HbA1c, glycated hemoglobin; SD, standard deviation

^1^ (date of diabetes diagnosis - date of birth + 1)/365.25; ^2^(date of consent – date of diabetes diagnosis + 1)/365.25); ^3^sitting position after 5 min of rest

*Data available for 122 patients

**Data available for 120 patients

SMBG measurements included the following: fasting SMBG was measured daily during the study alongside the 8-point SMBG profile: pre-breakfast, 2-h post-breakfast, pre-lunch, 2-h post-lunch, pre-dinner, 2-h post-dinner, bedtime, and at 3:00 AM. Patients were requested to perform 8-point SMBG profiles over a single 24-hour period on one day during the week before Visit 1 (baseline), at Week 4 (by phone call), at Week 8 (by phone call), at Week 12 (Visit 3), and at Week 24 (Visit 4). On days when 8-point profiles were done, fasting SMBG was considered as the first point of measurement, i.e., the pre-breakfast time point. Whenever the patient felt hypoglycemic symptoms, blood glucose had to be measured by the patient, referred to as SMBG during episodes of symptomatic hypoglycemia.

### Study outcomes

The primary efficacy outcome was the change in HbA1c from baseline to Week 24. For the eligibility and efficacy assessments of the study, HbA1c was measured at a laboratory agreed by the study site (certified level I “National Glycohemoglobin Standardization Program” laboratory) [15]. Secondary efficacy outcomes were mean HbA1c change from baseline to Week 12; mean change in fasting plasma glucose (FPG) from baseline to Week 12 and Week 24; mean change from baseline in fasting SMBG at Week 8, Week 12 and Week 24; mean change in 8-point SMBG from baseline to Week 12 and Week 24; the proportion of patients achieving HbA1c target of < 7.0% at Week 12 and Week 24; the proportion of patients achieving HbA1c target of < 7.0% at Week 12 and Week 24 without hypoglycemia (documented < 70 mg/dL) during the last 4 weeks of treatment; the proportion of patients achieving HbA1c improvement of at least 0.3% from baseline to Week 24 without nocturnal hypoglycemia and/or severe hypoglycemia (between 00.00 and 05:59 am SMBG) during the last 4 weeks of treatment; the proportion of patients with any improvement in HbA1c from baseline to Week 24 and decrease in occurrence of nocturnal hypoglycemia evaluated during the 4-week run-in period and the last 4 weeks on-treatment period; the proportion of patients with no deterioration in HbA1c from baseline to Week 24 and decrease in occurrence of nocturnal hypoglycemia evaluated during the 4-week run-in period and the last 4 weeks on-treatment period; the proportion of patients with no deterioration in HbA1c from baseline to Week 24 and no increase in occurrence of nocturnal hypoglycemia.

Safety endpoints were assessed by the number and proportion of patients experiencing hypoglycemia, as well as the number of hypoglycemic events per patient-year during the 4-week run-in period, the first 8 weeks of Gla-300 treatment, during the 24-week on-treatment period, and during the last 4 weeks on-treatment period. Hypoglycemic events were to be recorded on patient’s diary, according to definition and to the time occurrence. In addition to hypoglycemia events, adverse events (AEs)/Serious AEs (SAEs), and Product Technical Complaints (PTCs), vital signs, body weight and Gla-300 dose, were also recorded as part of the safety assessment.

Hypoglycemia was characterized as (1) symptomatic, confirmed ≤ 70 mg/dL and < 54 mg/dL; (2) severe, when an event necessitated the assistance of another person to actively administer carbohydrate, glucagon, or other corrective actions, confirmed ≤ 70 mg/dL and < 54 mg/dL; and (3) according to the time (nocturnal defined as time between 00.00 and 05:59 AM, and at any time of the day).

The effect of Gla-300 on patient-reported outcomes was evaluated by the Diabetes Treatment Satisfaction Questionnaire status (DTSQs) [16] and the Adult Low Blood Sugar Survey [17], which were collected during screening, V1, V3 (week 12), and end of treatment (week 24). Finally, patient satisfaction with the number of injections per day, and the importance for the patient of decreasing the number of basal insulin injections, were assessed on a 7-point Likert scale.

### Statistical analysis

A sample of 98 evaluable individuals was needed to detect a clinically relevant reduction in Hb1Ac of 0.3% with 85% statistical power, using a two-sided paired t-test at the 0.05 significance level with the estimate for the standard deviation (SD) of 0.98%. Assuming a dropout rate of 20%, the sample size required was 123 individuals.

Continuous data were summarized using the number of available data, mean, SD, median, minimum, and maximum. Categorical and ordinal data were summarized using the number and percentage of individuals. Parameters such as demographic and other baseline characteristics were summarized in the intent-to-treat (ITT) population. The completed population was composed by patients treated for a least 165 days and who had not permanently discontinued the study treatment before Week 24. The primary efficacy endpoint was analyzed using a mixed-effect model with repeated measures (MMRM) approach, under the missing-at-random framework carried out via PROC MIXED using an adequate contrast at Week 24. The mean change from baseline to Week 24 was accompanied by its 95% confidence interval (CI). Two statistical methods were used: paired Student’s t test comparing baseline and Week 24 values and HbA1c values as covariate. All secondary endpoints were analyzed or summarized on the 24-week treatment period using the ITT population. A similar analysis as performed for the primary efficacy endpoint was applied for the change in FPG and the change in SMBG. The proportions of patients experiencing hypoglycemia during the last 4 weeks on treatment and during the 4-week run-in period were analyzed using the McNemar’s test. Time points were compared using the Friedman’s test followed, when significant, by two-by-two Wilcoxon’s tests.

## Results

### Baseline characteristics

**Fig. 2:**
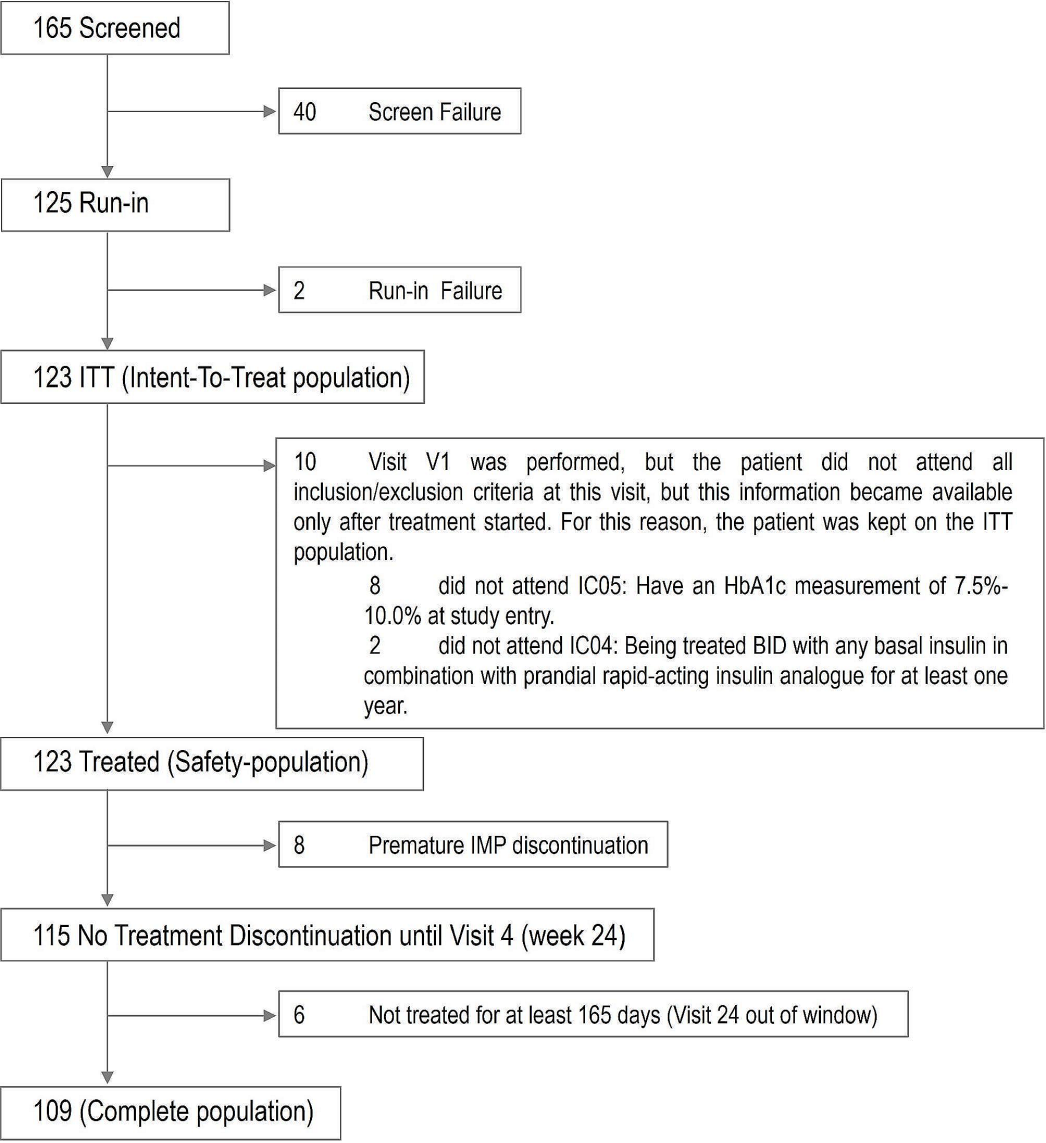
Patient flow with regards to eligibility and analysis

Of 165 people with T1D who signed the informed consent and were screened from 11 Brazilian Diabetes Centers, 40 were screening failures; among the latter, 34 individuals did not meet the inclusion criteria of an HbA1c measurement between 7.5% and 10.0% at study entry. Tertiary level Diabetes Centers were included after selection visit of institutions with previous experience in clinical studies, which presented the infrastructure and capacity to adequately conduct the present study. All eligible people with T1D were offered the study participation and were consecutively randomized. The run-in period was performed by 125 patients with T1D, two of which considered screening failures because the target accrual had already been reached. The ITT population was thus comprised of 123 individuals, and 109 people with T1D constituted the completed population (Fig. 2). Table 1 displays selected characteristics of the ITT population. Sixty-seven (54.5%) individuals were female, the mean (± SD) age was 37 ± 11.5 years, age at diagnosis was 17 ± 9.5 years and diabetes duration was 20.0 ± 9.8 years.

HbA1c and FPG at screening were 8.6 ± 0.7% and 201 ± 80.3 mg/dL, respectively. At baseline, 67%, 19%, 12% and 2% of patients were using Gla-100, detemir, isophane and degludec, respectively. The total daily basal insulin dose was 33.8 ± 14.3 U. Lispro was the prandial insulin used by most patients at the beginning (*N* = 60 [49%]) and end (*N* = 50 [41%]) of the run-in period. The total prandial daily insulin dose at these same time points was 26.6 ± 14.0 U and 25.1 ± 13.2 U, respectively.

Supplement Table 2 displays other characteristics of the study ITT population.

**Table 2:** Analysis of HbA1c from baseline to Week 24 in the study populations

	*N*	LSM^4^ estimate mean	LSM SE	95%CI	*p*-value
**HbA1c change from baseline to Week 24 (%)** ^**1**^					
ITT population	123	0.013	0.084	-0.153 to 0.179	0.8736
24-week on-treatment (ITT)^2^	123	0.024	0.084	-0.142 to 0.189	0.7755
Completed (ITT)^3^	109	-0.024	0.084	-0.191 to 0.143	0.7774

HbA1c, glycated hemoglobin; ITT, intent-to-treat, LSM, least square mean; SE, standard error

^1^based on a Mixed Model of Repeated Measures (MMRM) using the change from baseline to each post-baseline visit (Week 12 and Week 24) as the dependent variable and Visit as repeated measure fixed factor

^2^used in the model only HbA1c values performed until 7 days of the last dose of Gla-300 (Sensitivity analysis)

^3^model with the patients who completed the 24-week on-treatment period (at least 165 days of treatment) (Sensitivity analysis)

^4^Least Square Mean (obtained from the MMRM)

### Changes in basal and prandial insulin doses

There was a statistically significant increase in the mean total basal insulin dose from baseline to Week 24: least squares mean (LSM) estimate = 4.65 U; 95% CI, 2.95 to 6.34 U; *p* < 0.001. There was an increase in the mean total basal insulin dose from Week 0 to Week 24 of 4.72 ± 9.93 U and an increase of 0.06 ± 0.11 U/Kg/day between both visits. In terms of relative dose change, it was observed an increase of 18.31 ± 34.65% U and an increase of 17.12 ± 32.25% U/kg in basal insulin doses, from Week 0 to 24. For the prandial insulin dose, there was no statistically significant change from baseline to Week 24.

### Primary efficacy outcome

Table 2 displays results related to the change from baseline to Week 24 in HbA1c in the ITT population using the MMRM approach, alongside two sensitivity analyses (in the ITT population using only HbA1c values obtained within 7 days from the last dose of Gla-300 and in the completed population). In all cases, there were no statistically significant changes from baseline. Additional sensitivity analysis using paired Student’s t-test and MMRM with HbA1c value at baseline as a covariate, on the ITT population with all HbA1c values, ITT population only with on-treatment HbA1c values, and completed population, yielded similar non-significant results (data not shown).

### Secondary efficacy outcomes

Change in HbA1c from baseline to Week 12 was analyzed using the same MMRM approach, yielding no statistically significant results. Likewise, change in FPG from baseline to Weeks 12 and 24 did not reveal statistically significant differences. Change in SMBG from baseline to Weeks 8, 12 and 24 in the ITT population showed statistically significant decreases: at Week 8, LSM estimate= -10.23 mg/dL (95% CI, -18.06 to -2.40 mg/dL; *p* = 0.0109); at Week 12, LSM estimate= -17.78 mg/dL (95% CI, -25.90 to -9.66 mg/dL; *p* < 0.0001); and at Week 24, LSM estimate= -18.78 mg/dL; 95% CI, -26.67 to -10.89 mg/dL; *p* < 0.0001). The analysis of changes in 8-point SMBG from baseline to Weeks 12 and 24 showed statistically significant decreases for the time points pre-breakfast, post-breakfast and post-dinner, as well as a significant decrease from baseline to Week 24 for the time point pre-dinner (Table 3).

**Table 3:** Change in 8-Point SMBG from baseline to weeks 12 and 24

	*N*	LSM^2^ estimate mean	LSM SE	95%CI	*p*-value
**3:00 AM**					
SMBG change from baseline to Week 12 (mg/dL)	123	-14.493	8.902	-32.119 to 3.133	0.1061
SMBG change from baseline to Week 24 (mg/dL)	123	-15.078	7.807	-30.536 to 0.38	0.0558
**Pre-Breakfast**					
SMBG change from baseline to Week 12 (mg/dL)	123	-28.94	8.839	-46.437 to -11.442	0.0014
SMBG change from baseline to Week 24 (mg/dL)	123	-28.951	8.797	-46.365 to -11.538	0.0013
**Post-Breakfast**					
SMBG change from baseline to Week 12 (mg/dL)	123	-29.007	9.678	-48.167 to -9.846	0.0033
SMBG change from baseline to Week 24 (mg/dL)	123	-27.405	9.218	-45.655 to -9.156	0.0036
**Pre-Lunch**					
SMBG change from baseline to Week 12 (mg/dL)	123	-17.357	9.937	-37.028 to 2.313	0.0832
SMBG change from baseline to Week 24 (mg/dL)	123	-11.439	9.457	-30.16 to 7.282	0.2288
**Post-Lunch**					
SMBG change from baseline to Week 12 (mg/dL)	123	12.074	8.292	-4.342 to 28.49	0.1479
SMBG change from baseline to Week 24 (mg/dL)	123	-1.656	8.04	-17.573 to 14.261	0.8371
**Pre-Dinner**					
SMBG change from baseline to Week 12 (mg/dL)	123	-13.797	8.66	-30.94 to 3.347	0.1137
SMBG change from baseline to Week 24 (mg/dL)	123	-25.939	9.385	-44.518 to -7.361	0.0066
**Post-Dinner**					
SMBG change from baseline to Week 12 (mg/dL)	123	-19.152	9.138	-37.244 to -1.059	0.0382
SMBG change from baseline to Week 24 (mg/dL)	123	-19.977	9.122	-38.039 to -1.916	0.0305
**Bedtime**					
SMBG change from baseline to Week 12 (mg/dL)	123	-11.149	8.407	-27.795 to 5.497	0.1873
SMBG change from baseline to Week 24 (mg/dL)	123	-12.577	7.841	-28.103 to 2.949	0.1114

CI, confidence interval; LSM, least square mean; SE, standard error; SMBG, self-monitored blood glucose

Results based on a Mixed Model of Repeated Measures (MMRM) using the change from baseline to the post-baseline visits Week 12 and Week 24 as the dependent variable and Visit as repeated measure fixed factor

^2^Least Square Mean (obtained from the MMRM)

### Safety outcomes

Overall, 117 (95.1%) people with T1D reported a total of 2,920 hypoglycemic events. Table 4 displays mean differences in numbers of hypoglycemic events per patient-years between run-in and the last 4-week treatment period. Statistically significant decreases were observed for all events, symptomatic events, and confirmed ≤ 70 mg/dL events. There was a statistically significant decrease from run-in (78.0%) to the last 4 weeks on treatment (65.9%) in the proportion of patients with at least one hypoglycemic event (*p* = 0.0253). Body-weight changes from baseline (mean at end of run-in, 73.2 ± 13.2 Kg) to Week 12 (mean, 74.2 ± 13.3 Kg) and Week 24 (mean, 74.4 ± 13.5 Kg) were statistically significant for both time points (*p* = 0.0002 and *p* = 0.0008, respectively, using the MMRM approach).

**Table 4:** Mean differences in numbers of hypoglycemic events per patient-years between the run-in and last 4-week treatment periods

		Number of hypoglycemic events		Difference between the number of hypoglycemic events*		
	N with non-missing data	Run-in Mean (SD)	Last 4-weeks Mean (SD)	Mean (SD)	95%CI	*p*-value
**Hypoglycemic events by category**						
All	120	49.25 (52.23)	36.85 (45.08)	-12.40 (64.28)	-24.02 to -0.78	0.0367
Nocturnal	120	10.90 (19.63)	9.35 (18.39)	-1.55 (23.62)	-5.82 to 2.72	0.4737
Symptomatic	120	42.01 (46.25)	28.81 (40.02)	-13.21 (52.62)	-22.72 to -3.69	0.0069
Confirmed ≤ 70 mg/dL	120	47.30 (52.03)	35.66 (44.16)	-11.65 (64.06)	-23.23 to -0.07	0.0487
Confirmed < 54 mg/dL	120	18.71 (30.91)	13.37 (22.53)	-5.34 (37.07)	-12.04 to 1.37	0.1175
Severe	120	2.57 (11.99)	0.87 (4.70)	-1.70 (11.93)	-3.85 to 0.46	0.1221
Severe Confirmed ≤ 70 mg/dL	120	2.57 (11.99)	0.87 (4.70)	-1.70 (11.93)	-3.85 to 0.46	0.1221
Severe Confirmed < 54 mg/dL	120	1.95 (10.18)	0.87 (4.70)	-1.08 (10.74)	-3.02 to 0.86	0.2738

CI, confidence interval; n, number of patients with non-missing data; SD, standard deviation

* Calculated as the difference between number of events in ‘last 4-week treatment’ and ‘run-in’ periods

### Patient-reported outcomes

There was a statistically significant increase in total treatment satisfaction between baseline and both Week 12 and Week 24 (*p* < 0.0001 in both cases). The comparison among the time points Week 0 (baseline), Week 12 and Week 24 showed a statistically significant improvement of the score related to perceived hyperglycemia (*p* < 0.0001), with significant improvements between baseline and both Week 12 and Week 24 (*p* < 0.0001 in both cases). No significant differences were found for perceived hypoglycemia among the three visits. The evolution of HFS-II Behavior, Worry and Total scores revealed statistically significant improvement of the scores from baseline to Week 24 for HFS-II Behavior (*p* = 0.0361), HFS-II Worry (*p* = 0.0094), and HFS-II Total (*p* = 0.0051). The comparison of the patient satisfaction score (Question 1: Satisfaction with number of injections) at baseline (V1) and at Week 24 (V4) revealed a statistically significant improvement in the level of satisfaction (*p* < 0.0001).

## Discussion

In an out-of-target glycemic-control group of adult subjects with T1D, during this 28-week, multicenter, prospective, interventional, single-arm, open label phase 4 study, the switching from BID basal insulin to OD Gla-300 resulted in improved morning (pre- and post-breakfasting), late-afternoon (pre-dinner) and evening (pre- and post-dinner) capillary blood glucose levels. In addition, there was a decrease in overall hypoglycemia and an improvement in the level of satisfaction during this insulin treatment. There was no compromise in glycemic control as measured with HbA1c on the study period.

It is interesting to note that the majority (88%) of people with T1D were using basal insulin analogues at baseline in a developing country such as Brazil. Perhaps this can be explained by the fact that the protocol required patients to be on two daily doses of basal insulin, and most of the individuals in this study are from tertiary diabetic treatment centers where human insulin NPH as basal is given at least three times a day.

We found a small (around 5 U), but statistically significant, increase in the mean total basal insulin dose from baseline to the end of the study. These results reflect those from another study, in which people with T1D were randomized in an open-label fashion to Gla-300 or Gla-100 to morning or evening injection, continuing the mealtime analog, and followed for 6 months [18]. In that study, the basal insulin dose was 20% higher at 6 months, when insulin Gla-300 was given in the morning. The authors hypothesized that this finding could be a result of the up titration of Gla-300, as an attempt to maintain pre-breakfast glucose control. Another explanation to this increase could be a longer residence time of Gla-300 in the subcutaneous space and a higher enzymatic inactivation of this insulin by tissue peptidases at the injection site. This explanation was suggested by authors from another real-life study, in which patients with T1D were switched from Gla-100 to Gla-300 insulin, after 24 weeks, again with an observed increase in total basal insulin [19].

The analysis of 8-point SMBG from baseline to Weeks 12 and 24 showed a statistically significant decrease in pre- and post-breakfast and post-dinner, as in pre-dinner from baseline to Week 24. This finding is challenging, since two of the crucial points in the insulin treatment of patients with T1D are the dawn phenomena (reflex on fasting blood glucose) and the later-afternoon hyperglycemia (reflex on pre-dinner blood glucose). It is known that a considerable factor that contributes to poor glycemic control in T1D is the magnitude [9] and the prevalence [20] of the dawn phenomena.

Our finding of HbA1c reduction at the end of the follow-up in relation to the value presented at baseline, is similar to the results reported by Nakanishi et al. in an analysis of 20 patients with T1D who switched from Gla-100 to the same dose of Gla-300 for 3 months [21]. These authors found that the HbA1c levels were decreased in people with T1D, but not to a significant extent. In contrast, an open-label, real-world study conducted in the UK with 298 patients with T1D who had been receiving insulin Gla-300 for 6 months, demonstrated a mean significant reduction in HbA1c of 0.4% [22]. A statistically significant decrease in HbA1c during 24 weeks of treatment was also found in the OPTIMIZE study [23].

The TOP1 Trial was designed based on the OPTIMIZE study conducted by Mathieu et al. in Belgium and Canada, to assess the effect of treatment optimization with OD Gla-300 in combination with a prandial rapid-acting insulin in patients with T1D with HbA1c between 8 and 10% on BID basal insulin as part of basal-bolus therapy [23]. Since a statistically significant reduction in HbA1c from baseline to week 24 (mean difference 0.27%, *p* < 0.0001) was found in the OPTIMIZE trial, it was somehow disappointing that we were unable to observe the same result in our study population. However, despite the limitations of our study, we also found a decrease in the number of hypoglycemic events of all types in our study, that may have at least partly contributed to this difference in HbA1c results [24], as no significant effects on confirmed and/or severe hypoglycemia were observed in the Belgian and Canadian cohorts [23].

Overall, we found a decrease in the number of hypoglycemic events from run-in to last 4 weeks of treatment for events of all types and for symptomatic and confirmed (≤ 70 mg/dL) hypoglycemic events, even with the increased total basal insulin dose. It is important to remember that, in this trial, individuals with known hypoglycemia unawareness or with repeated episodes of severe hypoglycemia within the last 12 months were excluded. Other studies, as mentioned above [18], showed lower rates of overall hypoglycemia, and lower rates at night were shown only in the first 8 weeks. The last finding was also reported in a study from Japan which evaluated people with T1D using Gla-100 or Gla-300, with the latter having 38% less nocturnal hypoglycemia [25]. Our data, as the others studies [18, 25] with Gla-300, in relation to reduce the hypoglycemia prevalence was similar a recent study with another long action basal insulin (Degludec) in T1D [26].

In relation to body weight, our patients had a small (3.5%), but significant average gain during the study. This result differs from those of other studies in which there was either weight loss [18] or no change [22] when the basal insulin was changed to Gla-300. We believe the weight gain is associated with adjustment of the basal insulin dose without appropriate adjustment of the bolus dose. However, it is important to note that even with increased basal insulin and weight gain, there was a decrease in overall hypoglycemia rates and, at least, comparable efficacy.

In general, insulin Gla-300 was well tolerated, and a serious drug-related event occurred in 2.4% of the population, usually in the form of hypoglycemia. The frequency of AEs, TEAEs and events related to the application pen was low. No deaths were recorded during the study. There was no significant change in the laboratory parameters evaluated nor in the vital signs registered. Another important point in our study was the evaluation of quality of life, because this is considered to be directly linked to treatment adherence, and because hypoglycemic events can affect quality of life [27], in addition to the added cost burden [28]. Total satisfaction with treatment significantly improved between baseline and both Week 12 and Week 24, consistent with the findings of the OPTIMIZE study. The Evolution of HFS-II Behavior, Worry and Total scores also showed statistically significant improvement of the scores from baseline to Week 24. Finally, there was a statistically significant improvement in the level of patient satisfaction between baseline to Week 24.

There are several limitations in this study. The first is lack of complete adherence to *bolus* insulin optimization, which maintained a basal bolus disproportion and could explain the non-improvement of the SMBG lunch-period glycemic levels, while a limited number of patients were unable to show improvement in HbA1c despite improvement in other glycemic parameters. There is also the issue of “carry-over” between treatments, confounding the estimates of the treatment effect, as the run-in phase may not have been sufficiently long as a “wash-out” period between treatments. The strengths of our study are the continual assessment of glycemic profile, a study population from a developing country and comprised by difficult-to-treat patients, with long duration of T1D and out-of-targets HbA1c, and the use of patients as their own control.

## Conclusions

Switch from BID basel insulin to OD Gla-300 as part of basal *bolus* therapy in T1D resulted in similar glycemic control as measured by HbA1c, but provided significant improvements in SMBG, daily glucose profile, a lower incidence of hypoglycemia and increased patient satisfaction.

### Electronic supplementary material

Below is the link to the electronic supplementary material.

Supplementary Material 1

Supplementary Material 2

## Data Availability

The datasets are available from the corresponding author upon reasonable request.

## Abbreviations

AE: Adverse events
BID: Twice-daily
CI: Confidence interval
DTSQs: Diabetes Treatment Satisfaction Questionnaire status
FPG: Fasting plasma glucose
Gla-100: Insulin glargine 100 U/ml
Gla-300: Insulin glargine 300 U/mL
ITT: Intent-to-treat
LSM: Least squares mean
MMRM: Mixed-effect model with repeated measures
NPH: Neutral Protamine Hagedorn
OD: Once daily
PTCs: Product Technical Complaints
SAE: Serious AEs
SMBG: Self-monitoring of blood glucose
T1D: Type 1 diabetes
