# Supplementary material for: Effect of switching from twice-daily basal insulin to once-daily insulin glargine 300 U/mL (Gla-300) in Brazilian people with type 1 diabetes
Source: Diabetol Metab Syndr. 2024 Jul 9;16:152. doi: 10.1186/s13098-024-01385-x (PMC11232174; doi:10.1186/s13098-024-01385-x)
Supplement: Supplementary file 1 — Supplementary Material 1 [file 13098_2024_1385_MOESM1_ESM.pdf]

Internal

Supplement Table 1. Insulin glargine 300 U/mL (Gla-300) dose adjustment schedule.

**Median fasting[1] SMBG from last 3 days in the range of**	**Dose adjustment Gla-300 (unit/day)[2]**
>130 mg/dL (>7.2 mmol/L)	+1
Glycemic target: 70-130 mg/dL (3.9 to 7.2 mmol/L), inclusive	No change
<70 mg/dL (<3.9 mmol/L) or occurrence of ≥2 symptomatic or 1 severe hypoglycemia episode in the preceding week	-1 or at the discretion of the investigator

SMBG, self-monitoring of blood glucose

[1]Median is the middle value out of three values: two values from the two preceding days and one value from the current day.

[2]Dose adjustment should be done every 3 – 7 days.

Supplement Table 2. Baseline clinical characteristics of patients studied.

Characteristic (%)	ITT (N=123) N (%) or Value
**Gender**	*n=123*
Male	56 (45.5%)
Female	67 (54.5%)
**Age (years)**	*n=123*
Mean (SD)	37 (11.5)
**Age group (years)**	
18 - 29.9	37 (30.1%)
30 - 39.9	45 (36.6%)
40 - 49.9	19 (15.4%)
50 - 59.9	19 (15.4%)
≥60	3 (2.4%)
**Age group (years)**	
<65	122 (99.2%)
≥65	1 (0.8%)
**Patient considered as a smoking person[1]**	4 (3.3%)
**Frequency of alcohol intake during the last 12 months**	*n=123*
Daily	1 (0.8%)
Weekly	32 (26.0%)
Monthly	14 (11.4%)
Never	75 (61.0%)
Other ("Fortnight")	1 (0.8%)
**No. of standard drinks[2] containing alcohol on a typical day (count)**	*n=63*
Mean (SD)	2.2 (1.5)

**Age at diagnosis of diabetes[3] (years)**	*n=123*
Mean (SD)	17.0 (9.5)
**Duration of diabetes[4] (years)**	*n=123*
Mean (SD)	20.0 (9.8)
**Body weight (Kg)**	*n=122*
Mean (SD)	73.3 (13.2)
**Height (cm)**	*n=123*
Mean (SD)	166.9 (8.9)
**BMI (kg/m^2)**	*n=122*
Mean (SD)	26.3 (4.1)
**Systolic blood pressure[5] (mmHg)**	*n=123*
Mean (SD)	123.7 (12.8)
**Diastolic blood pressure[5] (mmHg)**	*n=123*
Mean (SD)	77.2 (9.3)
**Heart rate (beats per minute)**	*n=123*
Mean (SD)	79 (9.6)

N (%), number (percentage) of patients; n, number of patients with non-missing data; SD, standard deviation

[1]smoked at least 7 cigarettes per week, during last month; [2]standard drink=1 pint or bottle of beer, 1 glass of wine, 1 shot of hard liquor; [3](date of diabetes diagnosis - date of birth + 1)/365.25; [4](date of consent – date of diabetes diagnosis + 1)/365.25); [5]sitting position after 5 min of rest

FIGURE LEGENDS

Supplement Figure 1. Change in 8-Point SMBG from baseline to Weeks 12 and 24.
