# Supplementary figures and images for: Effect of switching from twice-daily basal insulin to once-daily insulin glargine 300 U/mL (Gla-300) in Brazilian people with type 1 diabetes
Source: Diabetol Metab Syndr. 2024 Jul 9;16:152. doi: 10.1186/s13098-024-01385-x (PMC11232174; doi:10.1186/s13098-024-01385-x)

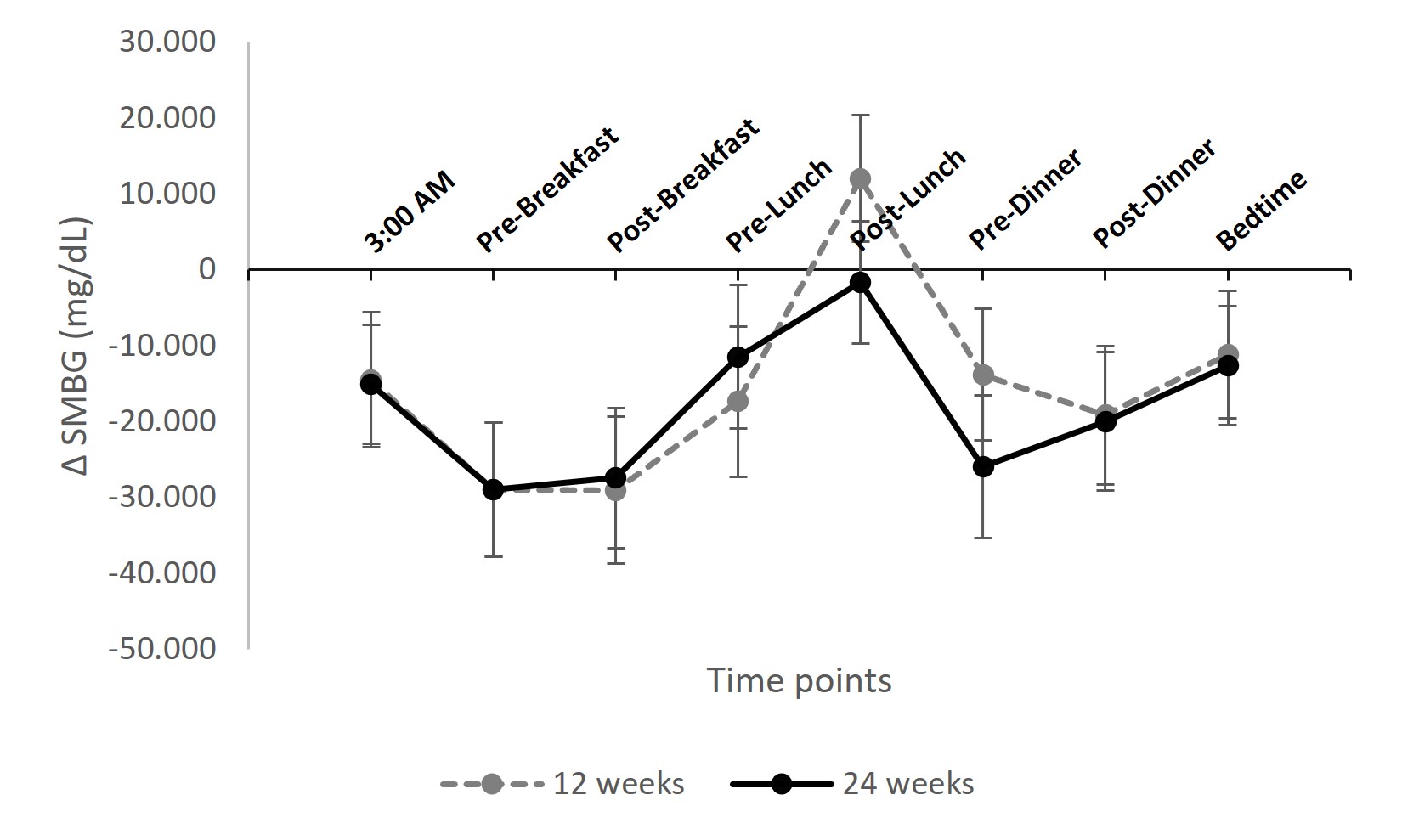

Supplement: Supplementary file 2 — Supplementary Material 2 [file 13098_2024_1385_MOESM2_ESM.jpg]
